# Can empowering leadership promote employees’ pro-environmental behavior? Empirical analysis based on psychological distance

**DOI:** 10.3389/fpsyg.2022.774561

**Published:** 2022-08-09

**Authors:** Ting Yue, Chenchen Gao, Feiyu Chen, Lan Zhang, Mengting Li

**Affiliations:** School of Economics and Management, China University of Mining and Technology, Xuzhou, China

**Keywords:** empowering leadership, psychological distance, green organizational climate, employees’ pro-environmental behavior, organizational management

## Abstract

Leadership styles, especially empowering leadership, affect the psychological relationship between employees and organizations, and then affect employees’ positive behavior in the organization. In this research, we studied the effects of empowering leadership and psychological distance on employees’ pro-environmental behavior and explored the mechanism of green organizational climate (GOC). By adopting correlation analysis, statistical analysis, and regression analysis, we conducted a multisource field study of 873 valid employee questionnaires to verify our theoretical model. The results showed that empowering leadership had a significant positive impact on psychological distance and employees’ pro-environmental behavior; and psychological distance played a partly intermediation role in the relationship between empowering leadership and employees’ pro-environmental behavior. Moreover, GOC can promote employees’ pro-environmental behavior, but it cannot regulate between psychological distance and employees’ pro-environmental behavior. The findings obtained some intriguing insights that could help to better guide employees toward pro-environmental behavior.

## Introduction

With the rapid development of China’s economy, the problem of resource shortage and environmental pollution becomes more and more serious, and environmental problem is getting more and more attention. Enterprises are both the largest social economic organizations in resource consumption and the largest social agents of pollution emission. For this reason, they must assume the social responsibility to protect the environment advocated by society and government (Liu, 2014). At the macro level, enterprises should accelerate technological innovation and transform into green development. And at the micro level, environmental practices of enterprises need the response and support of employees. The active participation of employees can greatly improve the environmental governance ability of enterprises (Afsar et al., 2016). Therefore, effectively guiding employees to behave well in pro-environmental behavior has become an urgent problem to be solved.

Employees’ pro-environmental behavior is divided into daily work-related pro-environmental behavior and daily spontaneous pro-environmental behavior (Bissing-Olson et al., 2013). Ones and Dilchert (2012) defined employees’ pro-environmental behavior as “all measurable behaviors in which employees actively participate that contribute to environmental sustainability or protect the environment from damage.” Lu et al. (2016) defined employees’ pro-environmental behavior as the positive and proactive environment-friendly behavior showed by employees in enterprise management practice. In general, employees’ pro-environmental behavior refers to the friendly behavior that employees spontaneously reduce or eliminate the negative impact in the work environment and strive to create a positive impact on the environment.

Leadership behavior plays a crucial role in effectively guiding employees’ pro-environmental behavior. A large number of research results have shown that positive leadership style positively affects employee behavior (Islam et al., 2020; Wang et al., 2020). Since the 21st century, the external environment has become increasingly dynamic and complicate, organizations are flattening their organizational structures and implementing employees self-management to adapt to the external environment. In that way, empowering leadership has attracted the attention of scholars for the advantage of improving employees’ self-leadership ability (Gavin, 2019). Relevant studies have suggested that empowering leadership is a positive and effective way of leadership, which can make employees feel that they are valued and trusted, and thus willing to take full advantage of their initiative and stimulate the vitality of the organization. Empowering leadership, as an important positive leadership style, has been proved to have a positive impact on employee behavior, such as organizational citizenship environmental behavior (Jiang et al., 2019), employee innovation behavior (Jada et al., 2019), employee safety behavior (Lee et al., 2019), and so on. Although relevant studies have reached relatively consistent conclusions, previous studies have not explained whether empowering leadership influences employees’ pro-environmental behavior and what the influencing mechanism is. Hence, it is necessary to further understand the relationship between empowering leadership and employees’ pro-environmental behavior. This research established a model of the influence of empowering leadership and employees’ pro-environmental behavior. Relevant studies have pointed out that empowering leadership affects employee psychological perception (Spreitzer, 1995), and the existence of perceived distance affects individuals’ organizational identification, which in turn affects individual decision-making behavior (Wang et al., 2013). Psychological distance refers to the subjective feeling of far and near (close and distant) relationship between people, as well as the perception of the emotional fit between people and organizations (He and Zhang, 2020). In addition, Li and Chen (2019) appealed that paying more attention to the emotional state of employees and the degree relationship between employees and the organization has a momentous significance in theory and practice for management research. This article introduces psychological distance, a psychological variable, to explore the potential mediating mechanism of empowering leadership affecting employees’ pro-environmental behavior and conduct relevant empirical research.

Except for the influence of social relationship, organizational environment is also very important for individual behavior guidance (Greenberg, 1988). For individual environmental behavior, green organizational climate (GOC) is an important manifestation of the organizational environment. GOC can affect the motivation, attitude, belief, and value of employees in the organization (Tagiuri and Litwin, 1968), where enterprise environmental policies and practices may make the employees have green values (Chou, 2014). Research suggests that environment-friendly work climate may improve employees’ pro-environmental behavior in and out of the workplace (Hicklenton et al., 2019). GOC is a crucial element affecting employees’ pro-environmental behavior. Based on that, this study proposes that employees seem more likely to develop green values and increase the possibility of achieving pro-environmental behavior in GOC.

Individual behavior depends on the interaction between individual perception and the external environment (Bandura, 1993). When external circumstances are extremely unfavorable or favorable, the behavior may be hindered or facilitated. Psychological distance is an internal psychological factor, and the employee–organization psychological distance (EOPD) perceived by individual may affect his/her decision-making. However, as an environmental factor, green organization climate may strengthen or weaken the influence of psychological factor on environmental behavior. For instance, Chou (2014) noted that GOC can regulate the relationship between individual environmental norms and employees’ pro-environmental behavior. Based on former researchers’ works, this article further examines the moderating effect of GOC in the relationship between psychological distance and employees’ pro-environmental behavior.

The main contributions of this article are as follows: first of all, we adopted the EOPD proposed by Chen and Li, explore the mediating mechanism between empowering leadership and employees’ pro-environmental behavior, and dig deep into the psychological process of empowering leadership’s influence on employees’ pro-environmental behavior. Second, green organization climate, as an environment variable, is examined for its effect on employees’ pro-environmental behavior and the external influence on employees’ pro-environmental behavior is further analyzed. Finally, this article explores the moderating role of GOC between psychological distance and employees’ pro-environmental behavior, which provides further empirical evidence for exploring the effect of empowering leadership on employees’ pro-environmental behavior in the context of green organization management.

## Theoretical basis and hypothesis development

### Empowering leadership and employees’ pro-environmental behavior

Employees’ pro-environmental behavior refers to the friendly behavior that employees consciously reduce or eliminate the negative impact and strive to create a positive impact on the environment at workplace. Proactive pro-environmental behavior can not only improve environmental conditions but also affect the environmental attitudes of organizations and individuals (Tian and Robertson, 2019). Employees’ pro-environmental behavior, as a kind of extra-role behavior, is altruistic. If the extra-role behavior is carried out, it means that the time for in-role behavior will be reduced (such as the time for doing their own work). Obviously, there is a conflict in the time allocation between extra-role behavior and in-role behavior. So it is particularly important for employees to break through the time allocation conflict from the bottom of their hearts and take the initiative to implement pro-environmental behavior.

Empowering leadership refers to a leadership style that shares power with staff by emphasizing the value of working, providing greater decision-making autonomy, expressing optimism about staff’s high performance, and removing barriers to performance (Zhang and Bartol, 2010). Facing the complex and changeable external environment, the organizational structure of enterprises is changing to a flattening in order to improve the adaptability of enterprises. In this context, empowering leadership, characterized by delegating power to employees and emphasizing employee participation, has gradually gained widespread attention in both theoretical and practical fields (Lee et al., 2018). And a wealth of studies have pointed out that empowering leadership has a positive impact on employee job performance, organizational citizenship behavior, employee creativity, employee satisfaction, and so on (Biemann et al., 2015; Dong et al., 2015; Cheong et al., 2016; Lee et al., 2018).

According to the theory of empowering leadership, this study argues that empowering leadership promotes employees’ pro-environmental behavior. First, empowering leadership helps employees better understand the meaning of their work and convinces them that their work and behaviors are crucial to organizational development. Second, empowering leadership helps employees gain competence and autonomy by encouraging employees to express their opinions and giving them greater decision-making autonomy and so on. Based on social exchange theory, employees will give feedback and rewards when they are motivated and trusted by their leaders. Specifically, leadership empowerment stimulates employees’ intrinsic motivation to contribute to the organization. Then employees will transcend role restrictions and spontaneously safeguard the interests of the organization to realize the corporate vision from the standpoint of the organization. Therefore, they are more inclined to do pro-environmental behavior that is conducive to the green development of the organization. Finally, empowering leadership also alleviates employees’ worries about their job challenges, which provides time for employees to do pro-environmental behavior. Based on this, we proposed the following hypothesis:

H1. Empowering leadership is positively related to employees’ pro-environmental behavior.

### The mediating role of psychological distance

The concept of psychological distance was initiated by psychologist Edward Bullough, meaning that esthetic feelings come from the psychological distance between the subjective sensation of the viewer and the observed object. Chen and Li (2018) applied this idea to the field of organizational management and proposed a new concept of “EOPD” and developed an EOPD scale. EOPD refers to a subjective judgment of the distance from which staff forecast, appraise, and act on the organization according to the real acceptance and the actual willingness to contribute. It is used to describe the fit between the staff and the organization. Consequently, drawing on the conceptual definition of Chen and Li (2018), psychological distance in this article specifically refers to the EOPD.

There are extensive studies on the relationship between leaders and psychological distance. Wade and Tavris (2002) proposed that when the leader reflected the employees’ psychological distance, job satisfaction and work efficiency would increase, otherwise, it was opposite. Then, Liberman et al. (2007) put forward that psychological distance would increase distally when leaders ignored employees’ survival and status or employees were threatened in the organization. Vanderstukken et al. (2019) suggested that if leaders could successfully evaluate psychological distance that subordinates may experience at some point, in that way they could readjust their communication methods, thereby exerting greater influence on subordinates. Moreover, Berson et al. (2015) found that the closer the psychological distance between leaders and followers was, the more followers would improve their promises and efforts to achieve personal aims and group aims. Li and Chen (2019) measured the intimacy level of the employee–organization relationship and proposed that managers should strengthen psychological and emotional intimacy with subordinates. This study believes that empowering leadership has an impact on psychological distance, mainly in the following several ways: (1) By helping employees understand work meaning and encouraging employees to make decisions autonomously, employees would have a better understanding of their work and a higher degree of participation, thus enhancing spatial-temporal distance. (2) Empowered employees would improve self-efficacy and have a good expectation for the future development of the organization. Experience distance would be closer. (3) Empowering leadership makes staff understand the value of work, has confidence in employees’ high performance, and helps them improve or accumulate working knowledge. Based on this, employees are closer to the organization emotionally and behaviorally, thus enhancing their emotional and behavioral distance. (4) With the help of empowering leadership, employees can better understand the company’s mission and values, and their personal values are more likely to converge the organization. The cognitive distance and objective social distance would be drawn closer. Hence, this article suggests that empowering leadership can narrow the psychological distance between employees and organizations. Accordingly, we proposed the following hypothesis:

H2. Empowering leadership is positively related to psychological distance.

Employees’ pro-environmental behavior is the result of the interaction of organizational environment, environmental awareness, and environmental motivation. Psychological distance involves the perception of the dynamic relationship between employees and the organization (He and Zhang, 2020). Therefore, this article believes that psychological distance is closely related to employees’ pro-environmental behavior. Specifically, (1) when the experiential distance, cognitive distance, and spatial-temporal distance between employees and the organization are closer, employees would have a clearer understanding of the future development of the organization and understand the significance of their behaviors to the organization’s development, so they are more likely to engage in pro-environmental behavior. (2) When the emotional distance between employees and the organization is closer, positive subjective cognition would stimulate employees’ subjective initiative, and employees are more likely to transcend role restrictions to implement pro-environmental behavior. (3) When the objective social distance between employees and the organization is relatively close, organization’s members are familiar with and identify with each other, which is conducive to the communication of internal members of the organization, forming the same green values, and then stimulating the production of pro-environmental behavior. (4) Pro-environmental behaviors are conducive to organizational development. When the behavioral distance between employees and the organization is closer, employees are more likely to adopt pro-environmental behaviors. In addition, Li and Chen (2019) proposed that EOPD could directly express the intimacy of the actual relationship between employees and the organization, and the smaller the psychological distance is, the more likely OCB occurs. Considering employees’ pro-environmental behavior as one of the concrete forms of OCB (Tian et al., 2019), we proposed that reducing psychological distance can promote the employees’ pro-environmental behavior. Hypothesis 3 is thus proposed as follows:

H3. A reduced psychological distance is positively related to employees’ pro-environmental behavior.

In the elaboration of H2 and H3, this study proposes the evidence and logic that empowering leadership can reduce psychological distance and promote employees’ pro-environmental behavior, as well as reducing psychological distance can promote employees’ pro-environmental behavior. This suggests that psychological distance may play a bridge role in the impact mechanism of empowering leadership on employees’ pro-environmental behavior. Mzembe’s (2020) study pointed out that the explanatory level of the cognitive object can be changed by changing individual’s psychological distance, thereby affecting the individual’s decision-making behavior. This suggests that in the mechanism of promoting employees’ pro-environmental behavior, empowering leadership can obtain the matching relationship between them by changing the psychological distance. The empowerment and incentive measures of empowering leadership reduce the psychological distance between employees and the organization. Employees tend to take high-level explanations that it is a good opportunity for personal growth, and their positive perceptions and expectations cause positive valence, which motivates them to participate in organizational behavior (Islam et al., 2021). Therefore, employees are more likely to engage in pro-environmental behaviors that benefit the organization. In addition, Cao et al.’s (2021) study also found that psychological distance plays a mediating role in the mediation of stress and employees’ innovative behavior. Thus, we proposed that psychological distance plays a mediating role between empowering leadership and employees’ pro-environmental behavior.

H4. Psychological distance strengthens the relationship between empowering leadership and employees’ pro-environmental behavior.

### Green organizational climate and employees’ pro-environmental behavior

Organizational climate is an employees’ shared perception regarding working environment, particularly formal policies, and guidelines (Norton et al., 2012). Recently, more and more researchers have begun to study different types of organizational climates, such as safety climate, service climate, innovation climate, and social climate. Chou (2014) developed the concept of GOC and proposed that GOC was perceived as a subclass of the organizational climate, which mainly includes green commitments of enterprise social responsibility, green organizational culture, and enterprise environmentalism.

A study has demonstrated that organizational climate noticeably affects staff’s emotions, attitudes, and behaviors in terms of work environment (Abdulkarim, 2014). The bulk of empirical studies has examined the impact of organizational climate on employees’ behavior. Wang and Peng (2018) proposed that pro-environmental passion climate was an emotional driving force, which made members enthusiastic about environmental actions, and had more driving force and predictive power for actual behaviors. Van der Werff et al.’s (2021) research indicated that the environmental impact of organizations and government would influence pro-environmental behaviors of citizens and employees. Based on the above research, we believe that GOC will affect employees’ decisions and behaviors. If employees perceive that they are in a green and environment-friendly organizational climate that encourages environmental behaviors, they are more inclined to implement pro-environmental behavior. Therefore, we hypothesized that:

H5. Green organizational climate is positively correlated with employees’ pro-environmental behavior.

### The moderating role of green organizational climate

Aside from the direct effect of GOC, we hypothesized that GOC may have a positive moderating effect on psychological distance and employees’ pro-environmental behavior. Norton et al. (2012) hypothesized that environmentally friendly organizational atmosphere moderated positive relationship between employees’ green knowledge, skills, abilities, and other personal factors and their green behavior. Chou (2014) verified that GOC could regulate the relationship between personal norms and employees’ pro-environmental behavior. Therefore, we hypothesized that GOC may regulate the relationship between psychological distance and employees’ pro-environmental behavior. When green organization climate is strong, the effect of psychological distance on employees’ pro-environmental behavior is stronger. Also, GOC can make employees feel the organization’s environmental protection enthusiasm, make them consistent with the values and goals of the organization, shorten the psychological distance, and thus promote employees to implement pro-environmental behaviors. On the contrary, when GOC is weak, the impact of psychological distance on employees’ pro-environmental behavior is weakened. Accordingly, we proposed the following hypothesis:

H6. Green organizational climate plays a moderating role in psychological distance and employees’ pro-environmental behavior.

**FIGURE 1:**
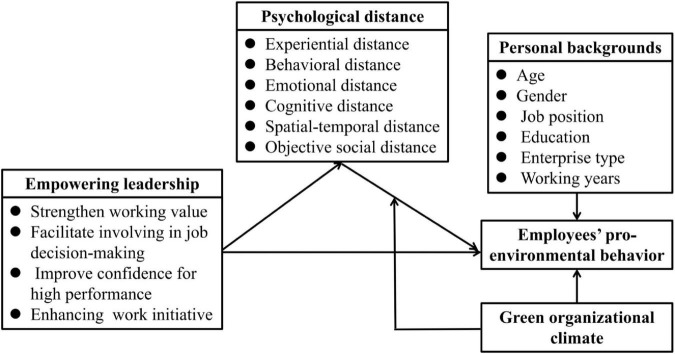
Hypothesis model.

In conclusion, a hypothesis model is proposed, as shown in Figure 1.

## Methodology

### Respondents and procedure

The research samples of this study were taken from 10 enterprises and institutions in Jiangsu province and Shandong province of China. To improve the respondents’ understanding of the content of the questionnaire as much as possible, a pre-research was conducted before the formal research. And the questionnaire items were revised according to the reliability and validity analysis results of the pre-research and the suggestions of the respondents. At the same time, this study adopted various methods such as anonymous answering, designing reverse questions, and random allocation of items of different dimensions to reduce the impact of public method bias on the research results.

In the pre-research stage, the questionnaires were distributed through social platforms such as WeChat, and employees were invited to fill in questionnaires. After excluding invalid questionnaires with too short answer time, wrong answers to reverse questions, or the basically same answers for measurement items, 183 valid questionnaires were obtained. The questionnaire items were revised according to the results of the pre-research and the suggestions of the research subjects, and the final formal questionnaire was formed. In the formal research stage, we chose to distribute paper questionnaires to MBA students in our college and asked 10 MBA students to send a link to the online questionnaires to their workgroup to obtain more reliable questionnaires. Finally, altogether 973 questionnaires were obtained, and 873 valid ones were obtained after eliminating the unqualified ones, with an effective rate of 89.72%. The participant’s demographic profile in Table 1 shows that the age, gender, education level, position, enterprise type, and working years of the respondents have a wide distribution and good representation.

**TABLE 1:** Participants’ demographic profile.

Item	Category	Frequency	%
Gender	Male	401	45.9
	Female	472	54.1
Age	18–25 years	95	10.9
	26–30 years	374	42.8
	31–40 years	232	26.6
	41–50 years	122	14
	51 years or more	50	5.7
Education	Junior college or below	134	15.4
	Bachelor	689	78.9
	Master or above	50	5.7
Position	General staff	602	69
	Front-line manager	144	16.5
	Middle manager	64	7.3
	Senior manager	41	4.7
	Others	22	2.5
Enterprise type	Government	116	13.3
	Public institution	126	14.4
	State-owned enterprise	136	15.6
	Private enterprise	178	20.4
	Foreign-capital enterprise	95	10.9
	Joint-stock enterprise	114	13
	Others	108	12.4
Working years	Under 1 year	176	20.2
	1–3 years	243	27.8
	4–6 years	199	22.8
	7–9 years	158	18.1
	Over 10 years	97	11.1

Data were collected using the questionnaires written by enterprise employees. The entire survey process was divided into two parts, namely, the pre-research and formal research. The former was mainly carried out through the online questionnaires. According to the pre-research results, the final formal questionnaire was formed by revising questionnaire items. At formal research stage, the paper questionnaires and the online questionnaires were mainly used. Finally, altogether 973 questionnaires were obtained, and 873 valid ones were obtained after eliminating the non-conforming ones, with an effective rate of 89.72%. In data analysis, the reverse questions were deleted according to the research need.

### Measures

This study collected data of five variables, namely, empowering leadership, psychological distance, employees’ pro-environmental behavior, GOC, and personal backgrounds. A five-point Likert scale was adopted in this article ranging from “strongly disagree (1)” to “strongly agree (5)”. The measures of the data of these five variables are as follows.

#### Empowering leadership

The empowering leadership scale developed by Ahearne et al. (2005) was used. This scale consists of 12 questions, including four dimensions: strengthening the value of working, facilitating involving in job decision-making, improving employees’ confidence for high performance, and enhancing their work initiative. Confirmatory factor analysis suggested that the four-factor model of empowering leadership had a good structural validity (χ^2^ = 128.485, df = 29, *p* < 0.001, NFI = 0.978, CFI = 0.971, RMSEA = 0.063). Moreover, Cronbach’s α was 0.938. An example item was “My superior will help me understand the correlation between my goals and company’s goals.”

#### Psychological distance

The scale developed by Chen and Li (2018) was adopted, consisting of 24 items, which was divided into six dimensions. Confirmatory factor analysis displayed that the six-factor model of psychological distance had a good structural validity (χ^2^ = 604.980, df = 174, *p* < 0.001, NFI = 0.962, CFI = 0.938, RMSEA = 0.053). Furthermore, Cronbach’s α was 0.972. An example item was “During this time at work, I have been well acquainted with the organization.”

#### Employees’ pro-environmental behavior

We used six items from Robertson and Barling’s (2013) scale to measure this variable, and Cronbach’s α was 0.813. An example item was “I will put recyclables (such as cans, paper, bottles, batteries, etc.) in the recycling bin.”

#### Green organizational climate

Green organizational climate developed by Taiwan scholar Chou (2014) was adopted and revised, and the final subscale included eight items. Cronbach’s α was 0.927. An example item was “Our company requires employees to give priority to environmentally friendly products when purchasing office supplies.”

#### Personal backgrounds

Respondents’ age, gender, job position, education, enterprise type, and working years were investigated in the first part of the questionnaire.

## Results

### Descriptive statistics and confirmatory factor analysis

Table 2 offers means, standard deviations, and correlations of the variables in the study. As shown in Table 2, there is a positive correlation between the variables: empowering leadership and employees’ pro-environmental behavior (*r* = 0.229, *p* < 0.01); empowering leadership and GOC, psychological distance (*r* = 0.164, *p* < 0.01; *r* = 0.142, *p* < 0.01); psychological distance and GOC, employees’ pro-environmental behavior (*r* = 0.230, *p* < 0.01; *r* = 0.127, *p* < 0.01); GOC and employees’ pro-environmental behavior (*r* = 0.221, *p* < 0.01).

**TABLE 2:** The means, standard deviations, and correlations of each variable.

	Variables	Means	Standard deviations	1	2	3	4
1	Empowering leadership	3.811	0.890	1			
2	Employees’ pro-environmental behavior	3.827	0.948	0.229**	1		
3	Green organizational climate	3.762	1.005	0.164**	0.221**	1	
4	Psychological distance	3.818	0.881	0.142**	0.127**	0.230**	1

**p < 0.01.

As empowering leadership, employees’ pro-environmental behavior, psychological distance, and GOC were collected from the same source (employees), we first tested the discriminative validity of the four variables through confirmatory factor analysis before testing research hypotheses. The results indicated (as shown in Table 3) that the four-factor model (χ^2^ = 2,296.350, df = 773, *p* < 0.01, NFI = 0.918, CFI = 0.944, RMSEA = 0.048, SRMR = 0.048) provided an obviously better fit than three-factor model (△χ^2^ = 1,061.057, △df = 3, *p* < 0.001), two-factor model (△χ^2^ = 5,740.327, △df = 5, *p* < 0.001) and one-factor model (△χ^2^ = 19,280.295, △df = 6, *p* < 0.001), and had a good matching data. The test results of confirmatory factor analysis indicated that the four research variables evaluated by employees, namely, empowering leadership, psychological distance, GOC, and employees’ pro-environmental behavior, have good discrimination validity.

**TABLE 3:** Summary of model fit indexes.

Model	Factor	χ^2^	*df*	△χ^2^	△*df*	NFI	CFI	RMSEA	SRMR
Model 1	Four-factor: PD; EL; GOC; and EPEB	2,296.350	773			0.918	0.944	0.048	0.048
Model 2	Three-factor: PD; EL; and GOC + EPEB	3,357.407	776	1,061.057	3	0.881	0.906	0.062	0.081
Model 3	Two-factor: PD + EL and GOC + EPEB	8,036.677	778	5,740.327	5	0.682	0.701	0.110	0.187
Model 4	One-factor: PD + EL + GOC + EPEB	12,576.645	779	19,280.295	6	0.553	0.568	0.132	0.221

*N* = 873, PD, psychological distance (six dimensions); EL, empowering leadership (four dimensions); GOC, green organizational climate (eight items); EPEB, employees’ pro-environmental behavior (six items); + means that two factors combine into one factor.

### Model test of mediating effect

First of all, model 4 in SPSS macro compiled by Hayes (2013) was adopted to test the intermediary role of psychological distance in the relationship between empowering leadership and employees’ pro-environmental behavior under the control of gender, age, education, enterprise type, job position, and working years. The results (as shown in Tables 4, 5) indicated that empowering leadership had a distinct predictive effect on employees’ pro-environmental behavior (β = 0.227, *t* = 6.381, *p* < 0.001). Moreover, when the intermediate variable was put into the middle, the direct predictive effect of empowering leadership on employees’ pro-environmental behavior was still significant (β = 0.214, *t* = 5.990, *p* < 0.001). Also, empowering leadership positively affected psychological distance significantly and psychological distance had a positive influence on employees’ pro-environmental behavior actively (β = 0.133, *t* = 3.940, *p* < 0.001; β = 0.096, *t* = 2.689, *p* < 0.01). In addition, upper and lower limits of 95% confidence intervals of the direct effect of empowering leadership on employees’ pro-environmental behavior and the mediating effect of psychological distance do not contain 0 (as shown in Table 5), indicating that psychological distance played a mediating role in the relationship between empowering leadership and employees’ pro-environmental behavior. The direct effect and the mediating effect accounted for 94.273 and 5.727% of the total effect, respectively.

**TABLE 4:** Mediation model test of psychological distance.

Regression equations (*N* = *873*)		Fit index			Coefficient significance	
Outcome variables	Predictive variables	*R*	*R* ^2^	*F*(*df*)	β	*t*
Employees’ pro-environmental behavior		0.252	0.063	8.386***		
	Gender				−0.059	−0.940
	Age				0.074	2.307*
	Education				−0.056	−0.800
	Enterprise type				0.001	0.036
	Job position				−0.006	−0.189
	Working years				0.021	0.800
	Empowering leadership				0.227	6.381***
Psychological distance		0.173	0.030	3.818***		
	Gender				−0.005	−0.075
	Age				0.053	1.764
	Education				−0.052	−0.780
	Enterprise type				0.005	0.349
	Job position				0.054	1.766
	Working years				0.005	0.190
	Empowering leadership				0.133	3.940***
Employees’ pro-environmental behavior		0.267	0.071	8.295***		
	Gender				−0.059	−0.936
	Age				0.069	2.150*
	Education				−0.051	−0.731
	Enterprise type				0.0001	0.004
	Job position				−0.011	−0.351
	Working years				0.020	0.785
	Psychological distance				0.096	2.689**
	Empowering leadership				0.214	5.990***

*p < 0.05;**p < 0.01; ***p < 0.001.

**TABLE 5:** Breakdown table of total effect, direct effect, and mediating effect.

	Effect value	Boot standard error	(Boot) LLCI	(Boot) ULCI	Relative effect value
Total effect	0.227	0.039	0.152	0.306	
Direct effect	0.214	0.040	0.135	0.295	94.273%
Mediating effect of psychological distance	0.013	0.007	0.003	0.028	5.727%

Besides, according to the analysis results, age could positively predict employees’ pro-environmental behavior significantly (β = 0.074, *t* = 2.307, *p* < 0.05).

### Model test of moderated mediating effect

Using Model 14 in SPSS macro compiled by Hayes (2013), the moderated mediating model was tested while controlling gender, age, education, enterprise type, job position, and working years. The results (as shown in Table 6) indicated that when GOC was included in the model as a moderating variable, the product of psychological distance and GOC (β = −0.022, *t* = −0.714, *p* > 0.05) had no significant predictive effect on employees’ pro-environmental behavior, indicating that GOC did not regulate the relationship between psychological distance and employees’ pro-environmental behavior. However, we could see that GOC had a notable positive prediction influence on employees’ pro-environmental behavior (β = 0.243, *t* = 2.015, *p* < 0.05).

**TABLE 6:** Moderating effect test of green organizational climate (GOC).

Regression equations (*N* = 873)		Fit index			Coefficient significance	
Outcome variables	Predictive variables	*R*	*R*2	*F*(d*f*)	β	*t*
Employees’ pro-environmental behavior		0.314	0.098	9.408***		
	Gender				−0.049	−0.787
	Age				0.061	1.912
	Education				−0.045	−0.648
	Enterprise type				0.002	0.137
	Job position				−0.013	−0.397
	Working years				0.017	0.649
	Empowering leadership				0.191	5.344***
	Psychological distance				0.143	1.158
	Green organizational climate				0.243	2.015*
	Psychological distance × Green organizational climate				−0.022	−0.714

*p < 0.05; ***p < 0.001.

## Conclusion

In this study, 873 employee questionnaires were used to investigate the influence of empowering leadership on employees’ pro-environmental behavior and the mechanism between them. The results indicated that both empowering leadership and EOPD had significant positive impacts on pro-environmental behavior, and psychological distance played an intermediary role between empowering leadership and employees’ pro-environmental behavior. In addition, organizational environment is an important factor influencing employees’ individual behavior, so we explored the impact of GOC on employees’ pro-environmental behavior. The results manifested that GOC could promote employees’ pro-environmental behavior, while GOC could not modulate the relationship between psychological distance and employees’ pro-environmental behavior. In a word, most of the hypotheses of this study have been confirmed.

### Theoretical implications

This study has important theoretical significance for future work on leadership style and employees’ pro-environmental behavior. In this research, we introduced psychological distance to explore the relationship between empowering leadership and employees’ pro-environmental behavior. Employees’ pro-environmental behavior in the workplace is an active environmental behavior at the individual level, so employees can choose freely whether to carry out the action or not. Furthermore, if employees choose pro-environmental behavior, it would not increase their own benefits, and may even cause inconvenience to work and increase work costs. If not, they also would not lose their own benefits (Zhao et al., 2018). Therefore, it is essential to guide employees to actively implement pro-environmental behavior. When EOPD is closer, the level of alignment or integration between staff and the organization is better (Chen and Li, 2018). Our research is consistent with its indication, which denotes that psychological distance performs the mediating functions between empowering leadership and staffs’ pro-environmental behavior. The employee whose psychological distance is closer to the organization will have more pro-environmental behavior. Employees’ pro-environmental behavior is an extension and specificity of organizational citizenship behavior (Tian et al., 2019). Our research results support the view proposed by Li and Chen (2019), that is, the closer the psychological distance is, the easier OCB occurs. Furthermore, our study has stretched the area of psychological distance and confirmed important implication of psychological distance in the organization and management field, hoping to provide some references for future research in the management field.

Although recent literature regarding the influencing mechanism of leadership style on employees’ pro-environmental behavior has emerged successively, the research regarding the influence of empowering leadership on employees’ pro-environmental behavior has not received much attention. Our study fills the gap in this area. Apart from that, recent studies have also found that the effectiveness of empowering leadership has not been universally supported (Wang and Sun, 2019), even though many studies have supported the positive role of empowering leadership. Our study provides the support of the experiment for the view on the behavior availability of empowering leadership.

Green organizational climate, as a relatively new concept, has less relevant research. Although the direct role of GOC is not the key issue of our study, we found that GOC can promote the employees’ pro-environmental behavior. Norton et al. (2012) pointed out that organizational environmental climate could help to understand how employees’ green behavior was created and encouraged in the workplace. In other words, there is a certain correlation between organizational environmental climate and employee’s green behavior in their workplace. This study offers empirical support for this view and verifies that GOC can stimulate employees’ pro-environmental behavior.

Besides, GOC cannot be used as a moderator between the relationship of psychological distance and employees’ pro-environmental behavior, which is inconsistent with our research hypothesis. It can be seen from the connotation of psychological distance that psychological distance is a comprehensive judgment result after the individual perceives external things. And it may result in that the level of individual psychological distance measured in this study is a perceptual result formed when other external factors are taken into consideration. In other words, GOC is more likely to be the antecedent variable of psychological distance, and the connection between psychological distance and GOC will be the subject of future research.

### Management implications

According to the above research conclusions, some advice is put forward for companies to correctly guide employees’ pro-environmental behavior: First, it is feasible to implement empowering leadership in the organization’s green development. Our study indicates that empowering leadership promotes employees’ pro-environmental behavior. Therefore, organization managers can adopt an empowering style to motivate employees’ pro-environmental behavior. Second, the implicit relationship between employees and organizations poses a huge challenge to organizational management, and organizations need to pay special attention to the EOPD. Construal level theory suggests that psychological distance determines people’s construal levels of things. That is, employee’s psychological distance to the organization will affect employee’s organizational identification, work contribution, and future expectations. In an organization, the occurrence of extreme events, abnormal dimensions, job burnout, and other phenomena are specific manifestations of EOPD alienation, which may lead to huge losses for the organization (Chen and Li, 2018). Therefore, the organization and leaders need to focus on EOPD, regular assessment EOPD, and take some measures (e.g., communicating more frequently with employees, helping employees to improve skills, improving employees’ self-efficacy, and work wellbeing), which are favorable to the development of organization. Third, the results indicate that GOC can facilitate the generation of employees’ pro-environmental behavior. On this account, we suggest that organizations should strengthen the development of internal green climate. For instance, developing environmental policy, purchasing green office supplies, organizing environmental lectures, and so on.

### Research limitations and prospects

This research does have some limits. First, we adopted a comprehensive score of psychological distance rather than an in-depth study of each dimension of psychological distance. The latter method can more specifically analyze the role of different dimensions of psychological distance in empowering leadership and employees’ pro-environmental behavior. Consequently, prospective researchers can consider the influence of various dimensions of psychological distance as an intermediary between empowering leadership and employees’ pro-environmental behavior.

Second, this study used a single point-in-time method to collect data, and a self-report method was used to measure all the variables involved, so common method bias is worthy of attention. Although we used a pre-controlled method to inform participants that there were no right or wrong answers, and to ensure the anonymity of participants, this would reduce the common method bias to some extent. We suppose that longitudinal study methods can be selected and more objective survey methods can be adopted to reduce this bias in the future.

Finally, some of the principles used in the study come from the West, while the tests come from China, which may not have universal research conclusions. In the future, employees’ pro-environmental behavior can be studied from the aspects of organizational culture and other aspects in the context of China’s national conditions.

## Data availability statement

The original contributions presented in the study are included in the article/supplementary material, further inquiries can be directed to the corresponding authors.

## Ethics statement

Ethical review and approval was not required for the study on human participants in accordance with the local legislation and institutional requirements. Written informed consent for participation was not required for this study in accordance with the national legislation and the institutional requirements.

## Author contributions

TY designed the frame and wrote the manuscript. CG collected and analyzed the data and wrote the manuscript. FC wrote the manuscript. LZ analyzed the data. ML collected the data. All authors contributed to the article and approved the submitted version.

## References

[B1] Abdulkarim R. M. (2014). *The Relationship Between A Leader’s Self-Perceived Level of Emotional Intelligence and Organizational Climate, As Perceived by Organizational Members.* Dissertation. Phoenix, AZ: Grand Canyon University.

[B2] Afsar B. Badir Y. Kiani U. S. (2016). Linking spiritual leadership and employee pro-environmental behavior: The influence of workplace spirituality, intrinsic motivation, and environmental passion. *J. Environ. Psychol.* 45 79–88. 10.1016/j.jenvp.2015.11.011

[B3] Ahearne M. Mathieu J. Rapp A. (2005). To empower or not to empower your sales force? An empirical examination of the influence of leadership empowerment behavior on customer satisfaction and performance. *J. Appl. Psychol.* 90 945–955. 10.1037/0021-9010.90.5.945 16162066

[B4] Bandura A. (1993). Perceived self-efficacy in cognitive development and functioning. *Educ. Psychol.* 28 117–148. 10.1207/s15326985ep2802_3

[B5] Berson Y. Halevy N. Shamir B. Erez M. (2015). Leading from different psychological distances: A construal-level perspective on vision communication, goal setting, and follower motivation. *Leadersh. Q.* 26 143–155. 10.1016/j.leaqua.2014.07.011

[B6] Biemann T. Kearney E. Marggraf K. (2015). Empowering leadership and managers’ career perceptions: Examining effects at both the individual and the team level. *Leadersh. Q.* 26 775–789. 10.1016/j.leaqua.2015.03.003

[B7] Bissing-Olson M. J. Iyer A. Fielding K. S. Zacher H. (2013). Relationships between daily affect and pro-environmental behavior at work: The moderating role of pro-environmental attitude. *J. Organ. Behav.* 34 156–175. 10.1002/job.1788

[B8] Cao Y. Zhou H. Gu J. (2021). The impact of work stress on employees’ innovation behavior: Mediating effect of psychological distance and moderating effect of employment relationship atmosphere. *Sci. Sci. Manag. S& T* 42 163–176.

[B9] Chen H. Li S. S. (2018). Measuring the psychological distance between an organization and its members—The construction and validation of a new scale. *Front. Psychol.* 8:2296. 10.3389/fpsyg.2017.02296 29375427 PMC5767263

[B10] Cheong M. Spain S. M. Yammarino F. J. Yun S. (2016). Two faces of empowering leadership: Enabling and burdening. *Leadersh. Q.* 27 602–616. 10.1016/j.leaqua.2016.01.006

[B11] Chou C.-J. (2014). Hotels’ environmental policies and employee personal environmental beliefs: Interactions and outcomes. *Tour. Manag.* 40 436–446. 10.1016/j.tourman.2013.08.001

[B12] Dong Y. T. Liao H. Chuang A. Zhou J. Campbell E. M. (2015). Fostering employee service creativity: Joint effects of customer empowering behaviors and supervisory empowering leadership. *J. Appl. Psychol.* 100 1364–1380. 10.1037/a0038969 25774571

[B13] Gavin M. (2019). *7 ways to empower your employees. Harvard Business Review.* Available online at: https://online.hbs.edu/blog/post/how-to-empower-employees

[B14] Greenberg J. (1988). Cultivating an image of justice: Looking fair on the job. *Acad. Manag. Exec. (1987-1989)* 2 155–158. 10.2307/4164817

[B15] Hayes A. F. (2013). Introduction to mediation, moderation, and conditional process analysis: A regression-based approach[J]. *J. Educ. Meas.* 51, 335–337.

[B16] He W. Y. Zhang M. T. (2020). Overqualification, psychological distance and employee’s innovative behavior: Cross-Layer moderation effect of corporate social responsibility. *Sci. Technol. Prog. Policy* 037 144–152. 10.6049/kjjbydc.2019030510

[B17] Hicklenton C. Hine D. W. Loi N. M. (2019). Can work climate foster pro-environmental behavior inside and outside of the workplace? *PLoS One* 14:e0223774. 10.1371/journal.pone.0223774 31600307 PMC6786752

[B18] Islam M. N. Furuoka F. Aida I. (2020). Transformational leadership and employee championing behavior during organizational change: The mediating effect of work engagement. *South Asian J. Bus. Stud.* 11 1–19. 10.1108/SAJBS-01-2020-0016

[B19] Islam M. N. Idris A. Furuoka F. (2021). The role of leadership to nurture employee championing behavior during organizational change: Does valence matter? An individual level analysis. *Employee Responsibil. Rights J.* 34 1–17. 10.1007/s10672-021-09375-1

[B20] Jada U. R. Mukhopadhyay S. Titiyal R. (2019). Empowering leadership and innovative work behavior: A moderated mediation examination. *J. Knowl. Manag.* 23 915–930. 10.1108/JKM-08-2018-0533

[B21] Jiang M. Q. Wang H. Y. Li M. Z. (2019). Linking empowering leadership and organizational citizenship behavior toward environment: The role of psychological ownership and future time perspective. *Front. Psychol.* 10:2612. 10.3389/fpsyg.2019.02612 31849746 PMC6895145

[B22] Lee A. Willis S. Tian A. W. (2018). Empowering leadership: A meta-analytic examination of incremental contribution, mediation, and moderation. *J. Organ. Behav.* 39 306–325. 10.1002/job.2220

[B23] Lee Y. H. Lu T. E. Yang C. C. Chang G. (2019). A multilevel approach on empowering leadership and safety behavior in the medical industry: The mediating effects of knowledge sharing and safety climate. *Saf. Sci.* 117 1–9. 10.1016/j.ssci.2019.03.022

[B24] Li S. S. Chen H. (2019). Closeness or distance? An investigation of employee–organization relationships: From a psychological distance perspective. *Front. Psychol.* 9:2765. 10.3389/fpsyg.2018.02765 30733699 PMC6353851

[B25] Liberman N. Trope Y. Strphan E. (2007). “Psychological distance,” in *Social psychology: handbook of basic principles*, eds Kruglanski A. W. Higgins E. T. (New York, NY: Guilford Press).

[B26] Liu J. Q. (2014). Research on environmental protection from the perspective of corporate social responsibility. *Adv. Mater. Res.* 869–870 714–717.

[B27] Lu H. Liu X. Chen H. (2016). The connotation, structure and measurement of enterprise employee pro-environmental behavior. *Soft Sci.* 30 69–74. 10.13956/j.ss.1001-8409.2016.08.16

[B28] Mzembe A. (2020). The psychological distance and construal level perspectives of sustainable value creation in SMEs. *Sustain. Dev.* 28 1–14. 10.1002/sd.2150

[B29] Norton T. A. Zacher H. Ashkanasy N. M. (2012). On the importance of pro-environmental organizational climate for employee green behavior. *Ind. Organ. Psychol.* 5 497–500. 10.1111/j.1754-9434.2012.01487.x

[B30] Ones D. S. Dilchert S. (2012). Environmental sustainability at work: A call to action. *Ind. Organ. Psychol.* 5 444–466. 10.1111/j.1754-9434.2012.01478.x

[B31] Robertson J. L. Barling J. (2013). Greening organizations through leaders’ influence on employees’ pro-environmental behaviors. *J. Organ. Behav.* 34 176–194. 10.1002/job.1820

[B32] Spreitzer G. M. (1995). Psychological empowerment in the workplace: Dimensions, measurement, and validation. *Acad. Manag. J.* 38 1442–1465. 10.2307/256865

[B33] Tagiuri R. Litwin G. (1968). *Organizational climate: Explorations of a concept.* Boston, MA: Harvard University Press.

[B34] Tian Q. Robertson J. L. (2019). How and when does perceived CSR affect employees’ engagement in voluntary pro-environmental behavior? *J. Bus. Ethics* 155 399–412. 10.1007/s10551-017-3497-3

[B35] Tian Q. Yang H. X. Peng D. F. Lv W. Y. (2019). How to improve organizational citizenship behavior for the environment? The mediation effect of altruistic concern and the moderation effect of organizational identification. *Hum. Resour. Dev. China* 36 24–36. 10.16471/j.cnki.11-2822/c.2019.02.002

[B36] Van der Werff E. Steg L. Ruepert A. (2021). My company is green, so am I: The relationship between perceived environmental responsibility of organizations and government, environmental self-identity, and pro-environmental behaviors. *Energy Effic.* 14:50. 10.1007/s12053-021-09958-9

[B37] Vanderstukken A. Schreurs B. Germeys F. Van den Broeck A. Proost K. (2019). Should supervisors communicate goals or visions? The moderating role of subordinates’ psychological distance. *J. Appl. Soc. Psychol.* 49 671–683. 10.1111/jasp.12626

[B38] Wade C. Tavris C. (2002). *Psychology*, 7th Edn. New York, NY: Prentice Hall, Pearson Publishing.

[B39] Wang D. S. Han J. Li T. T. (2020). How does the authentic leadership restrain the employees’ CWB? -Mediated by leader-member exchange and moderated by self-efficacy. *Res. Econ. Manag.* 41 134–144. 10.13502/j.cnki.issn1000-7636.2020.07.009

[B40] Wang H. L. Sun J. M. (2019). The negative effects of empowering leadership: Theoretical mechanisms and boundary conditions. *Adv. Psychol. Sci.* 027 858–870. 10.3724/SP.J.1042.2019.00858

[B41] Wang L. P. Yu Z. C. Wang S. H. (2013). Research on the influences of psychological distance on knowledge sharing behavior: The mediating role of perceived organizational support. *Sci. Sci. Manag. S& T* 34 37–45.

[B42] Wang Q. Y. Peng J. (2018). CEO Green Transformational leadership and corporate green behavior: The role of environmental responsibility culture and pro-environmental passion climate. *Hum. Resour. Dev. China* 35 83–93. 10.16471/j.cnki.11-2822/c.2018.01.008

[B43] Zhang X. Bartol K. M. (2010). Linking empowering leadership and employee creativity: The influence of psychological empowerment, intrinsic motivation, and creative process engagement. *Acad. Manag. J.* 53 107–128. 10.5465/AMJ.2010.48037118

[B44] Zhao S. Kang M. Wang M. (2018). The impact of benevolent leadership on employees’ pro-environmental behavior: The role of supervisor-subordinate Guanxi and power distance. *Stud. Psychol. Behav.* 16 101–108. 10.3969/j.issn.1672-0628.2018.06.013

